# Impact of an integrated whiteboard system on physics QA turnaround time

**DOI:** 10.1002/acm2.13703

**Published:** 2022-06-19

**Authors:** Evan Gates, Bing‐Hao Chiang, Alan Kalet, Minsun Kim

**Affiliations:** ^1^ Department of Radiation Oncology University of Washington Seattle Washington USA

**Keywords:** quality assurance, standardization, whiteboard

## Abstract

**Purpose:**

To evaluate the impact of a digital whiteboard system integrated with data from the oncology information system (OIS) on the urgency of physics quality assurance (QA) tasks in the radiation oncology department.

**Methods:**

Quality check list (QCL) items in the Mosaiq OIS corresponding to eight discrete, sequential steps in the treatment planning process were created. A whiteboard to graphically display active QCLs automatically and in real time was implemented in March 2020 using R shiny. QCL data with completion status were collected in two 12‐month time periods before and after whiteboard implementation: January 2019–December 2019 and July 2020–June 2021. For all plans requiring patient‐specific QA, we recorded when each plan was available for physics QA and which treatments started the following day. We further classified those plans into four categories (urgency levels 1–4 with 4 being the most urgent) depending on how much time was available to perform QA. We compared the proportion of these next‐day QAs in each category between time periods accounting for plan type, day of the week, and time of year.

**Results:**

Overall QA numbers were similar between time periods with 797 and 765 QAs total. The total proportion of next‐day QA decreased by 27% and the proportions of urgency levels 1 and 4 both showed significant decreases after whiteboard implementation of 29.2% and 54.9%, respectively (p<0.05). All plan types had reduced proportions of next‐day QAs, especially nonstereotactic body radiation therapy (non‐SBRT) (30.3% decrease, p<0.05). Fridays and the months of October–December had the highest proportion of next‐day QAs but showed significant reductions of 19.1% and 40.6% in the proportion of next‐day QAs, respectively (p<0.05).

**Conclusions:**

The integrated whiteboard system significantly reduced the proportion of last‐minute physics work, increasing patient safety. Advantages of the integrated whiteboard were low cost, low overhead with automatic interface to the OIS, and concurrent user support.

## 1 INTRODUCTION

Treatment planning in radiation oncology is a complex, sequential process that is usually constrained to be completed with a fixed time window. While much of the planning time is determined by discrete tasks like drawing contours, much time can be lost due to inefficient communication between individuals or groups.^1^ Delays in the planning process lead to challenging and urgent situations. These reduce the quality of patient care by increasing the chance of human error and decreasing quality of work.^2^ Each clinic establishes its own workflows and procedures with varying degrees of effectiveness in optimizing the timing of treatment planning steps. One method is the use of a whiteboard system that standardizes the planning process by defining and visually displaying discrete planning steps for each patient. Standardization of workflow has been recently discussed as a key aspect of improving treatment quality.^3^

Whiteboards have been shown to improve communication and safety in hospital settings like emergency and critical care.^4^, ^5^, ^6^ In these settings, success depends on the ability to manage resources and staff and adapt to changing demands. Whiteboards realize these goals by centralizing information and making it quickly available at a glance in real time. In radiation oncology specifically, electronic whiteboard systems have been successfully implemented in a handful of clinics with reductions in planning and contouring times.^7^, ^8^, ^9^ Explanations for these benefits include increased transparency and a reduced need for other formal communication.^10^ For physicist workflows specifically, a whiteboard was able to increase physics plan coordination.^11^ Many whiteboard implementations use in‐house solutions that build on existing data stores in the oncology information system (OIS). This can greatly reduce this cost while still maintaining the stability provided by commercial software. An in‐house solution also provides customized features that adapt to department‐specific workflows and preferences. Overall, whiteboards are natural, flexible tools for improving safety and efficiency in the radiation oncology clinic.

Physics quality assurance (QA) is at the end of the planning process and therefore, the available turnaround time is often the result of the cumulative time gains or losses in all the previous steps. For this reason, physics QA time may be a good surrogate for workflow efficiency and increases in QA time available may indicate improvements in the upstream processes. In this work, we quantitatively evaluated the impact of an integrated electronic whiteboard system on the time available for physics QA and plan checks.

## 2 METHODS

A whiteboard system integrated with the OIS was developed and deployed in the Department of Radiation Oncology at the University of Washington to standardize and streamline the department's workflow. The department treats approximately 1200 external‐beam radiotherapy patients per year using four linear accelerators. The integrated whiteboard system consists of two software components which were implemented concurrently: A quality check list items (QCLs) chain in the MOSAIQ (Elekta) OIS to represent the steps in the treatment planning process and a graphical whiteboard visualization developed in an R environment^12^ (R version 3.2.3; 10 December 2015) using the Shiny^13^ web‐based application library.

For the QCL chain, we identified eight serial steps in the treatment planning process and created QCLs corresponding to each step. For example, the first QCL in the process, 1 queue, is automatically generated when the patient completes simulation and a simulation note is generated by the simulation staff. Then, patient CT scans for treatment planning are transferred to the treatment planning system by a dosimetrist. Once this step is completed, they complete the first QCL, 1 queue, which automatically generates the next QCL, 2 contours. This second QCL goes to the physician alerting them that the patient is ready for their contours of tumor and organs‐at‐risk. This process continues until the last QCL, 8 rtt check, is completed when therapists complete their pretreatment check. The full list of QCLs used in our whiteboard steps and their descriptions are listed in Table 1. Medical physicists are responsible for two steps near the end of the process: 6 physics qa, which is patient‐specific, intensity‐modulated radiation therapy (IMRT) QA as applicable, and 7 physics check, an initial plan check performed for all patients. The creation time of the 6 physics qa QCL indicates when the plan is available for physics QA and checks.

**TABLE 1:** Descriptions of each step in the integrated whiteboard system and corresponding quality check list (QCL) items

QCL	Responsible	Description
1 queue	Dosimetrist	CT scan export to treatment planning system
2 contours	Physician	Contouring of target volumes and organs‐at‐risk
3 planning	Dosimetrist	Treatment plan generation
4 md approval	Physician	Review and approval of treatment plan
5 export	Dosimetrist	Plan and beam parameters export to OIS
6 physics qa	Physicist	Patient‐specific QA if needed
7 physics check	Physicist	Initial plan check
8 rtt check	Therapist	Final pretreatment check

**FIGURE 1:**
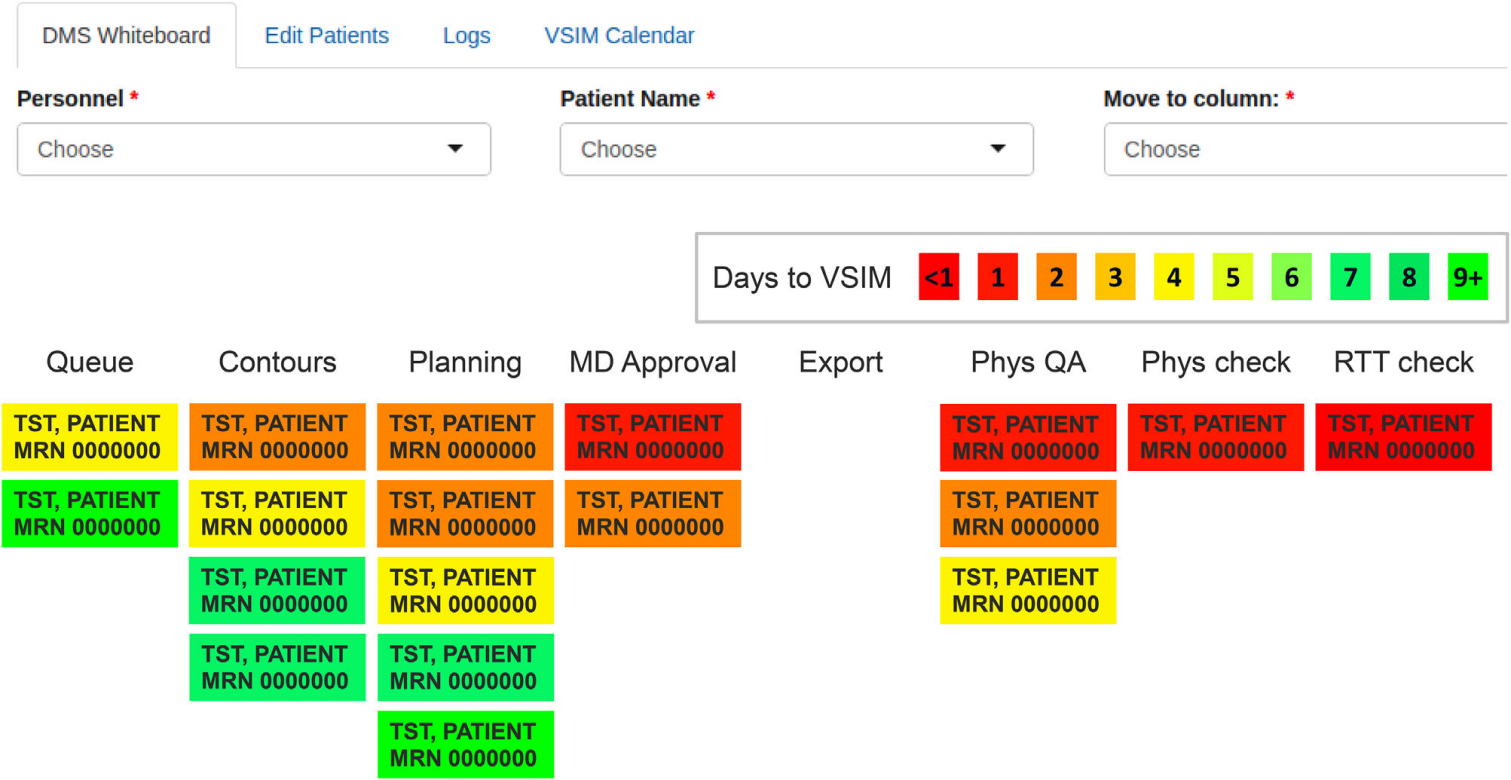
Schematic of the whiteboard application showing color‐coded cards representing patients

The whiteboard user interface displays a series of tables where a patient is represented by a card resting in the table column identifying which part of the planning process the patient is in. A partial screenshot of the whiteboard display is shown in Figure 1. The background of each patient card is color coded according to the number of days remaining before their predetermined start date, or virtual simulation (VSIM). Each patient's treatment planning process, from simulation to delivery, was tracked using automatically generated QCLs. When a process step QCL is completed by a user, the next QCL is automatically generated and sent to the responsible party (Table 1) via “IQ scripts” in the MOSAIQ OIS.^14^ IQ scripts allow the user to customize the workflows within MOSAIQ by building logical blocks. As QCLs are completed in the MOSAIQ OIS, the patient cards are automatically moved in the whiteboard via live queries of the OIS database. Thus, the whiteboard presents a visualization and real‐time update of where all patients are in the process and what their state of “urgency” is, in a way that is not possible in MOSAIQ QCL lists alone. Furthermore, because the whiteboard display queries QCL data, users only interact with the system by completing QCLs in MOSAIQ. This is advantageous because they do not need to access a separate system outside OIS.

The shiny application and server are used to create interactive forms.^15^ For our application, the hardware hosting the file structures and application code are encrypted and administered according to institutional IT & security criticality level 4, which is appropriate for resources that host Health Insurance Portability and Accountability Act of 1996 (HIPAA) regulated patient data. Initial logins are handled by authentication via Shibboleth: our instance of the single‐sign‐on identification software, and authorization by a system administrator. Users are allowed access according to local group permissions.^16^ With single‐sign‐on, users access to the whiteboard uses the same username and password that they use for other hospital computer systems meaning access is controlled without having to maintain a separate user database. Code for the integrated whiteboard system and app is available on request.

To evaluate the effectiveness of the integrated whiteboard system, we collected QCL data from two 12‐month periods surrounding the implementation date of March 2020: January 2019–December 2019 and July 2020–June 2021. These periods were intentionally selected to exclude the transitional period around the switch to the integrated whiteboard system while encompassing the same calendar periods. Data were collected for all patients requiring patient‐specific QA measurements. In our clinic, patient‐specific QA was performed daily using ArcCHECK^1^ after patient treatments were finished around 5:00 PM. We chose this population of cases to study since they have increased physics workload due to QA measurements, which are done during nonclinic hours. For these cases, a plan check generally cannot be completed on the same day as the plan is available because after‐hours QA must be completed first.

The creation dates and times of 6 physics qa QCLs were collected from QCL histories and centralized self‐reported logs completed by physicists. These logs were maintained for evaluating QA workload distribution among physicists and provided a cleaned version the necessary data for this study. An alternative would have been to query the MOSAIQ database directly and inspect the output but the resulting data would have been equivalent. However, using self‐reported logs has two advantages over an Structured Query Language (SQL) query. First, the records are already intelligently curated. This is especially important for patients with multiple treatment courses, replanned treatments, or multiple QCLs where the correct timestamp comparisons would be very difficult from a query alone. In other words, self‐reported logs more faithfully represent the actual relationships between QA availability and treatment start which is the focus of this study. Second, before the integrated whiteboard system there were no systematic QCLs to extract time stamps from so self‐reported logs are the only way to compare time periods. The collected data included each corresponding patient's treatment start date, start time, and treatment plan type. Plans were labeled as: step‐and‐shoot IMRT, volumetric modulated arc therapy (VMAT), and or stereotactic body radiation therapy (SBRT). QAs were labeled as “next‐day” (ND) if the patient's first treatment was the same or day or the day following generation of the 6 physics qa QCL. Weekends and holiday were not counted. Since QA cannot start before patient treatments are done, plans available for QA any time in the workday are effectively the same despite potentially several hours time difference. The next‐day binning captures this fact and is more useful than an absolute number of hours.

Among the next‐day QAs we designed, four different urgency levels‐based circumstances that put increased pressure on physicists in our clinic. The criteria are summarized in Table 2. But, the specific criteria defining these levels can be generalized for other clinics based on their particular workflow. The set of next‐day QAs with ample time on both days is urgency level 1 (lowest). QAs available late in the afternoon give reduced time for QA preparation and pose a slightly higher urgency, level 2. We used a cutoff time of 3:00 PM. to categorize these QAs in this study. Treatments starting next‐day and early the following morning (cutoff time chosen as 10:00 AM) create particularly urgent circumstances and place increased pressure on physicists for rapid initial plan check so we designated this subset as urgency level 3. The highest urgency situation (level 4) included the cases that satisfied the criteria of both level 2 and level 3.

**TABLE 2:** Urgency levels for QAs that put increased pressure on physics

Urgency level	QA required by next day	QA available after 3:00 PM	Patient treats before 10:00 AM
level 1	×		
level 2	×	×	
level 3	×		×
level 4	×	×	×

QA, Quality Assurance; IMRT, Intensity modulated radiation therapy; VMAT, Volumetric arc therapy; SBRT, Stereotactic body radiation therapy.

We compared the time periods before and after whiteboard implementation using the proportions of next‐day QAs and urgent QAs in each. We further compared these proportions within certain subsets. We compared next‐day QA proportions by day of the workweek and time of the year to investigate if there is any correlation between the time of the week or the time of the calendar year and the proportion of the next‐day QAs. We also compared the proportion of next‐day QAs for various plan types: (1) IMRT versus VMAT and (2) SBRT versus non‐SBRT, to investigate if QA time depends on plan types. Differences in the proportions were assessed for statistical significance using Fisher's exact test with a significance level of 0.05 (implemented in R version 4.1.0).^17^

## 3 RESULTS

The periods before and after whiteboard implementation showed similar overall workloads of patient‐specific QA with 797 and 765 plans, respectively. The proportion of next‐day QAs decreased from 46.9% to 34.4%, corresponding to nearly 100 fewer next‐day QAs in the 12‐month period. The proportion of next‐day QAs at the lowest urgency level, level 1, was reduced by 29.2%, which was a significant change (Fisher's exact test p<0.05). Similarly, the highest urgency level, level 4, also showed a large significant decrease in proportion by 54.9%. The proportion of QAs with urgency levels 2 and 3 also decreased between time periods but these differences were nonsignificant (22.5% and 41.8%, respectively). The number of QAs and proportions for both time periods are listed in Table 3.

**TABLE 3:** Number of urgent plans by time available for physics QA and treatment start time before and after whiteboard implementation. *p*‐Values are from Fisher's exact test on the proportions (ND: next day).

	Before: Jan 2019–Dec 2019		After: Jul 2020–Jun 2021		
Total QA	797		765		*p*
Total ND	374	46.9%	263	34.4%	<0.001
Urgency 1	184	23.1%	125	16.3%	<0.001
Urgency 2	122	15.3%	100	13.1%	0.39
Urgency 3	38	4.8%	25	3.3%	0.16
Urgency 4	30	3.8%	13	1.7%	0.01

QA, Quality Assurance; ND, next‐day.

**FIGURE 2:**
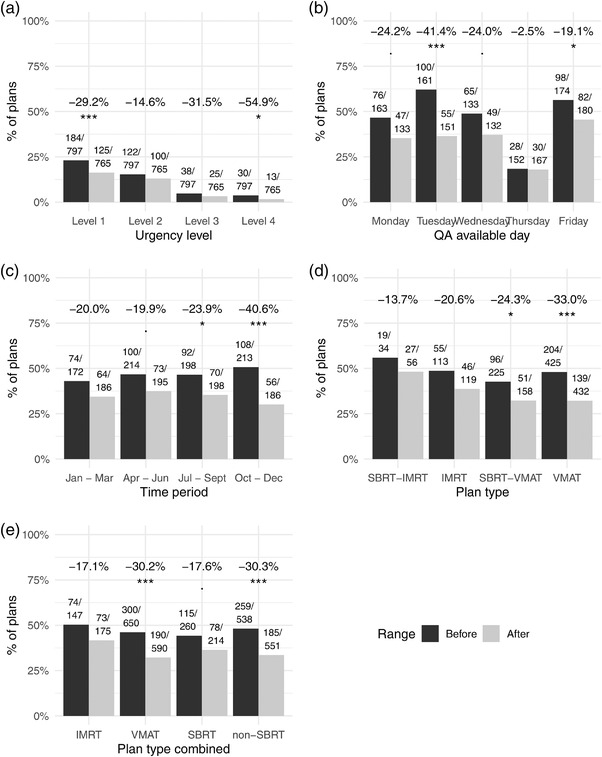
Proportional decrease in next‐day quality assurances (QAs) overall, for different urgency levels, and for relevant subsets. Each bar is the proportion of overall plans with the specific numbers listed above. Percentages above each pair of bars are the percent change in the proportion before and after whiteboard implementation. Significance is indicated: ***p<0.001, **p<0.01, *p<0.05, p<0.1

Proportions of next‐day QAs in general were not evenly distributed among the days of the work week as shown in Table 4. Before whiteboard implementation, QAs available on Tuesday were over three times as likely to be next‐day treatment as QAs available on Thursday (62.1% vs. 18.4%). After whiteboard implementation, the range of next‐day QA proportions was smaller with the lowest proportion of 18.0% on Thursday and 45.6% on Friday. The Tuesday rate was decreased to 36.4% which is consistent with the surrounding days. These differences are visualized in Figure 2b.

**TABLE 4:** Number of urgent plans separated by the day of the week they were available for QA. The values are plotted in Figure 2. QCLs available on weekends were excluded. *p*‐Values are from Fisher's exact test on the proportions of next‐day QAs.

	Before: Jan 2019–Dec 2019			After: Jul 2020–Jun 2021			
QA available day	Total	# ND	% ND	Total	# ND	% ND	*p*
Monday	163	76	46.6%	133	47	35.3%	0.06
Tuesday	161	100	62.1%	151	55	36.4%	<0.001
Wednesday	133	65	48.9%	132	49	37.1%	0.06
Thursday	152	28	18.4%	167	30	18.0%	1.00
Friday	174	98	56.3%	180	82	45.6%	0.04

QA, Quality Assurance; ND, next‐day.

Overall, the proportions of next‐day QAs decreased across every 3‐month time period, Table 5 and Figure 2c, with significant decreases in the July through September and October through December periods. Before whiteboard implementation, more than half of the QAs in the months of October through December were next‐day QA. This proportion was higher than any other 3‐month period in the year. After whiteboard implementation, the October through December period actually had the lowest proportion of next‐day QAs (30.1%) compared to other 3‐month periods.

**TABLE 5:** Number of urgent plans separated by time of year. The values are plotted in Figure 2c. *p*‐values are from Fisher's exact test on the proportions of next‐day QAs.

	Before: Jan 2019–Dec 2019			After: Jul 2020–Jun 2021			
Month block	Total	# ND	% ND	Total	# ND	% ND	*p*
Jan–Mar	172	74	43.0%	186	64	34.4%	0.10
Apr–Jun	214	100	46.7%	195	73	37.4%	0.07
Jul–Sept	198	92	46.5%	198	70	35.4%	0.03
Oct–Dec	213	108	50.7%	186	56	30.1%	<0.001

QA, Quality Assurance; ND, next‐day.

We also investigated the urgent plan proportions per plan type: IMRT, VMAT, IMRT–SBRT, or VMAT–SBRT. The proportion of overall plans in each category was comparable between time periods with roughly one‐third of cases being SBRT and one‐fifth of cases being IMRT (Table 6). All plan types showed a decrease in the proportion of next‐day QAs after the implementation of the whiteboard system with the biggest improvement seen in non‐SBRT plans and VMAT plans (Figure 2d,e). Both of these types had a significant 30% reduction in the proportion of next‐day QAs.

**TABLE 6:** Number of urgent plans separated by type of plan. The values are plotted in Figure 2d,e. *p*‐Values are from Fisher's exact test on the proportions of next‐day QAs

	Before: Jan 2019–Dec 2019			After: Jul 2020–Jun 2021			
Plan type	Total	# ND	% ND	Total	# ND	% ND	*p*
SBRT‐IMRT	34	19	55.9%	56	27	48.2%	0.52
IMRT	113	55	48.7%	119	46	38.7%	0.15
SBRT‐VMAT	225	96	42.7%	158	51	32.3%	0.04
VMAT	425	204	48.0%	432	139	32.2%	<0.001
SBRT	260	115	44.2%	214	78	36.4%	0.09
Non‐SBRT	538	259	48.1%	551	185	33.6%	<0.001
IMRT	147	74	50.3%	175	73	41.7%	0.14
VMAT	650	300	46.2%	590	190	32.2%	<0.001

QA, Quality Assurance; ND, next‐day; IMRT, Intensity modulated radiation therapy; VMAT, Volumetric arc therapy; SBRT, Stereotactic body radiation therapy.

## 4 DISCUSSION

We found that implementation of a structured electronic integrated whiteboard system significantly reduced the number of next‐day plan QA tasks in our department by 27%, which corresponds to over 100 plans a year. We saw a consistent decrease in next‐day QAs across time periods and plan types, which suggests this reduction is likely due to process improvement rather than a consequence of different workload characteristics between time periods. We attribute much of this improvement to the integrated whiteboard system's standardization of the hand‐off between planning steps. The specific QCLs help guard against ambiguity and information loss that can reduce the overall efficiency. On a larger scale, the whiteboard's visual interface helps team members plan ahead based on upcoming cases and prioritize cases they are responsible for. How each staff member specifically uses each component of the system is left to personal preference, for example, how often they choose to monitor the QCL chains or the graphical web page, or both. Ultimately, work is assigned and marked complete using the standard QCLs.

Before implementation of the whiteboard system, information about a patient's treatment plan progress was passed using nonstandardized and often informal means. These included ad hoc, nonstructured QCLs, emails, phone calls, and impromptu hallway communication. In our clinic, we noted some inefficiencies: First, inconsistency may create ambiguity or confusion about where (or how) to find information about the progress of a particular patient's plan. Second, phone calls and verbal communication do not create records so information may be forgotten. Furthermore, these methods generate no data which can be reviewed later to improve workflow. Third, it is difficult to manage multiple, concurrent tasks (e.g., treatment plans) at once under this paradigm since crucial information (like treatment start date) might be inconsistently located. These motivated the development and implementation of our electronic integrated whiteboard system.

The results presented agree with our experiences and impression about usual clinic workloads. Monday was the most frequent day for patients to start treatment (35% of all plans), which may explain why next‐day QAs were more frequently available on Fridays. New patients rarely start treatment on Fridays, which explains why next‐day QA available on a Thursday was least frequent. We saw the biggest improvement on Wednesday starts (QA available on Tuesdays) and suspect it may be because it is more feasible to improve the process efficiency during the middle of the week. Anecdotally, Fridays generally have more inconsistent staffing due to vacation, academic days, and so forth, right before the weekend.

We also noted that the improvement was most pronounced during the fourth quarter of the calendar year (October through December) compared to other times of the year. Anecdotally, complex scheduling and staffing availability during this holiday period often poses additional challenges to the treatment planning process and a whiteboard implementation could help improve the process. The proportion of next‐day QAs for IMRT plans was higher than for VMAT plans and was not significantly reduced by whiteboard implementation (17.1% decrease, p=0.14). In our clinic, we use step‐and‐shoot technique for IMRT when precise beam angles are needed to avoid critical structures like pacemakers or metal implants and their artifacts. Although, in general VMAT is preferred due to shorter a delivery time at the machine.

While we were able to reduce the proportion of urgent QAs using the whiteboard system, some of next‐day QAs may be unavoidable due to circumstances like urgent palliative treatment or midtreatment replanning. Calculating the lowest possible rate of next‐day QAs would require more detailed data collection on the entire treatment planning process and factoring in additional case‐specific details. These questions could be answered by future work mining the whiteboard QCL data for other process steps and identifying external factors like outside imaging, concurrent chemotherapy scheduling, and insurance authorization that influence the treatment planning timeline.

We focused on the turnaround time for physics QA as it may represent an overall assessment of the treatment planning workflow. Our results support increasing the scope of the analysis in future work to identify where the strongest gains and room for improvement are. In addition to analyzing the time spent in other whiteboard process steps, the results could be stratified by other factors that may modify treatment plan complexity and timing. These include attending physician (different physicians may preferentially treat different patient populations at larger centers), planning dosimetrist, treatment site, and treatment intent. These data could help to further refine the integrated whiteboard system to better meet the needs of the clinic.

## 5 CONCLUSION

We successfully developed and introduced a custom electronic whiteboard system integrated with the OIS in our clinical workflow. Implementation of the electronic whiteboard reduced the proportion of next‐day physics QAs by 27% at our clinic, which corresponds to over 100 plans in a 1‐year period. The proportion of next‐day QAs decreased across times of year, days of the workweek, and treatment plan types. Small differences in the rates of improvement were consistent with our understanding of our specific clinic. We conclude that integrated whiteboard systems are a straightforward way to improve workflow efficiency in radiation oncology departments.

## CONFLICT OF INTEREST

The authors declare that there is no conflict of interest that could be perceived as prejudicing the impartiality of the research reported.

## AUTHOR CONTRIBUTIONS

Conceptualization: Alan Kalet, Minsun Kim. Study design: Evan Gates, Bing‐Hao Chiang, Alan Kalet, Minsun Kim. Data collection and analysis: Evan Gates, Bing‐Hao Chiang, Minsun Kim. Figures: Evan Gates, Alan Kalet. Manuscript writing and revision: Evan Gates, Bing‐Hao Chiang, Alan Kalet, Minsun Kim.

## FUNDING INFORMATION

The authors received no specific funding for this work.

## Data Availability

Research data and code will be shared upon reasonable request to the corresponding author.

## Footnotes

1 Sun Nuclear Corporation, Melbourne, FL.
